# Cellulase Activity Screening Using Pure Carboxymethylcellulose: Application to Soluble Cellulolytic Samples and to Plant Tissue Prints

**DOI:** 10.3390/ijms15010830

**Published:** 2014-01-09

**Authors:** Hanne R. Johnsen, Kirsten Krause

**Affiliations:** Department of Arctic and Marine Biology, UiT The Arctic University of Norway, Dramsvegen 201, Tromsø N-9037, Norway; E-Mail: hanne.r.johnsen@uit.no

**Keywords:** carboxymethylcellulose, cellulase activity, Gram’s iodine staining, plant tissue prints

## Abstract

Reliable, rapid and inexpensive detection of cellulolytic enzymes that can be used for a wide variety of biological and environmental samples are currently in high demand. Here, a new cellulase detection protocol is described that circumvents problems observed with popular agar-based methods by exploiting the ability of carboxymethylcellulose (CMC) to form gel-like surfaces on its own. These pure CMC-layers are sensitive to cellulolytic degradation and stainable by Gram’s iodine without showing unwelcome reactions with other enzymes. The staining intensity negatively correlates with the enzyme activity and can be used for quantification. Cellulase activities are not obstructed by high sugar contents (e.g., in plant material) which limit the applicability of other quantification methods, making our new method particularly attractive for screening of plant extracts. A useful variant of this new method is its applicability to plant tissue prints for spatial mapping of the cellulolytic activity in a zymogram-like fashion.

## 1. Introduction

Plant cell walls consist of a network of interwoven biopolymers that form crystalline structures. The major cell wall polymer is cellulose, followed in abundance by hemicelluloses, lignin and pectins. Plant cell walls have long been recognized as a potential sustainable energy source, provided that their natural resistance to enzymatic degradation can be overcome. Enzymes catalyzing the break-down of cellulose have evolved naturally in a number of organisms feeding on living or decaying plant material [1–4] as well as in the plants themselves. These enzymes are of potential value for effectivity-maximization of biofuel production from plant debris [5]. Currently, a considerable variety of species and environments are screened for cellulolytic enzymes that possess improved characteristics, a process for which reliable detection and quantification of cellulase activity is a prerequisite.

Cellulolytic activity can be quantified by a variety of methods that have been summarized in recent papers [6,7]. Since crystalline cellulose is degraded at very slow rates, most assays were adapted to use more easily degradable soluble cellulose derivatives like carboxymethylcellulose (CMC) [8]. Screening for extracellular cellulase production by bacteria and fungi is often done on agar plates containing CMC as substrate [4]. The detection of the cellulolytic activity in these cases is achieved by staining or precipitation of undigested CMC in plate regions which were not exposed to cellulolytic activity, while areas exposed to cellulase give clear halos surrounding the source of the enzyme. This method is popular because large numbers of samples can be monitored and compared simultaneously and quickly. Over the decades, a variety of dyes have been introduced for this differential staining, the most common of which is Gram’s iodine [8,9].

Quantitative spectrophotometric assays based, for example, on 3,5-dinitrosalicylic acid (DNSA) are often used in addition to validate the outcome of the initial screen and to quantify the activity. In crude plant extracts, the spectrophotometric detection of cellulolytic activity, however, can be aggravated by several factors. Tissue that is rich in sugars creates high background in DNSA-based quantitative assays, largely eliminating it from this way of quantification. Chromophores like chlorophyll, carotenoids and anthocyanin as well as phenolic compounds can also interfere with spectrophotometric measurements. There is to date no single satisfactory method that allows the qualitative and quantitative measurements of cellulase activities from plant tissue.

The foremost aim of this study was to quantify cellulase activities from the parasitic weed *Cuscuta*. Controls that were performed during initial attempts revealed shortcomings of the CMC–agar plate assays, prompting us to search for a rapid and inexpensive alternative that fulfilled our need for a suitable assay for plant tissue without being limited to plants.

## 2. Results and Discussion

**Figure 1.**
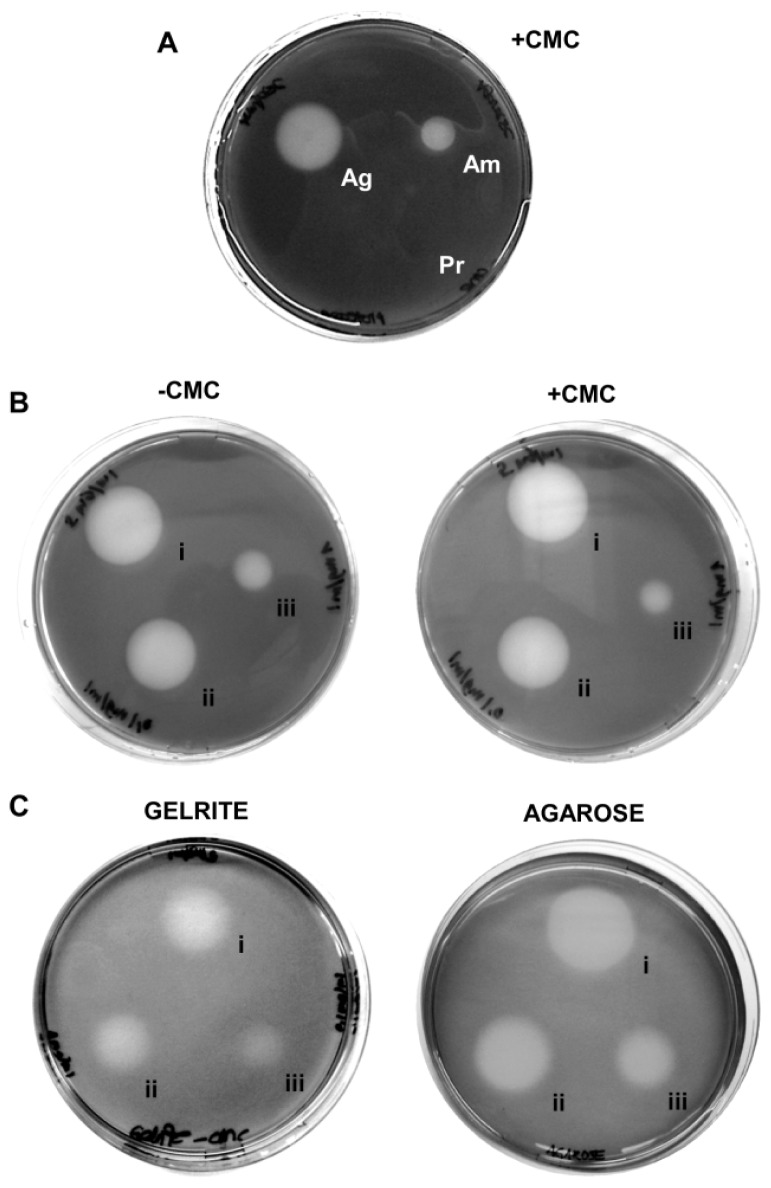
Conventional agar plate-based clearing assays with Gram’s iodine yield unwanted artifacts. (**A**) Unspecific halo occurrence with non-cellulolytic enzymes: 5 μg agarase (Ag), 5 μg amylase (Am) and 5 μg proteinase K (Pr) were spotted onto 1% agar with 0.2% CMC and stained with Gram’s iodine solution after incubation. Halos are visible around both agarase and amylase; (**B**) Unspecific halo occurrence in the absence of the substrate: 25 μg (i), 5 μg (ii) and 0 μg (iii) cellulase from *Aspergillus niger* were spotted onto agar with and without 0.2% CMC as cellulolytic substrate and detected by Gram’s iodine. Halo formation of comparable extent independent of the CMC can be seen; and (**C**) Unspecific halo occurrence on other gelling agents: Cellulase in the same concentrations as in (**B**) was spotted onto plates containing 0.2% CMC in 1% agarose or in 0.75% Gelrite and detected by Gram’s iodine. Halos are more diffuse than on agar, but can be clearly distinguished.

Despite its wide use, the plate clearing assay based on CMC–agar is notorious for its low specificity, producing halos around other polymer-degrading enzymes like amylase and agarase (Figure 1A), independent of the presence or absence of a cellulase substrate (e.g., CMC) (Figure 1B). The artifacts occur likewise with other gelling agents like agarose and Gelrite (Figure 1C) and with other staining methods like Congo Red (data not shown). Among these problems, particularly the substrate-independent formation of halos seriously impacts one of the most important criteria of enzyme assays, their specificity and precludes any chances to quantify cellulase activities. A quarter of a century ago, Zitomer and Eveleigh [10] already recommended to exercise caution when interpreting results using iodine stained CMC–agar plates. They found that small amounts of contaminating starch in commercial agars made the staining unreliable. Their concerns have been substantiated and extended now by the controls shown here (Figure 1). The use of CMC–agar (or –agarose or –Gelrite) in connection with Gram’s iodine staining as a stand-alone method can thus not be regarded as reliable.

**Figure 2.**
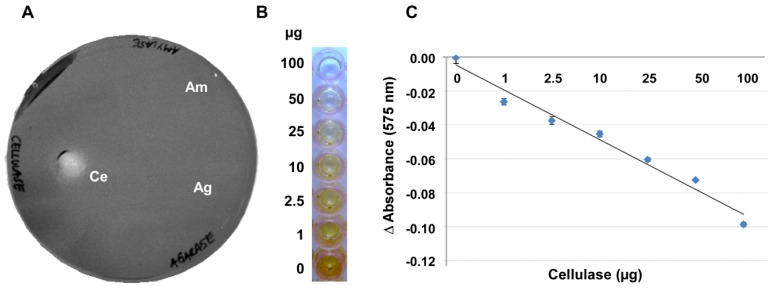
Cellulase activity can be detected on pure solidified CMC without unwanted artifacts. (**A**) Assay in petri dish format: Gram’s iodine detection of 5 μg cellulase (Ce), 5 μg amylase (Am) and 5 μg agarase (Ag) spotted onto 7% solidified CMC; and (**B**,**C**) Assay in 96-well plate format: Different amounts of cellulase from *Aspergillus niger* were applied to a gelatinous CMC-layer (4%) in the wells of a 96-well plate. Hydrolysis of the substrate after 16 h of incubation at 27 °C was measured photometrically at 575 nm after Gram’s iodine staining using a microtiter plate reader. Delta (Δ) absorbance values were obtained by deducting the absorbance values of the undigested control wells from those of the sample wells and were plotted against the amount of cellulase (in μg) applied to each well. Error bars in (**C**) indicate the deviation of the mean value of 13 repetitions.

**Figure 3.**
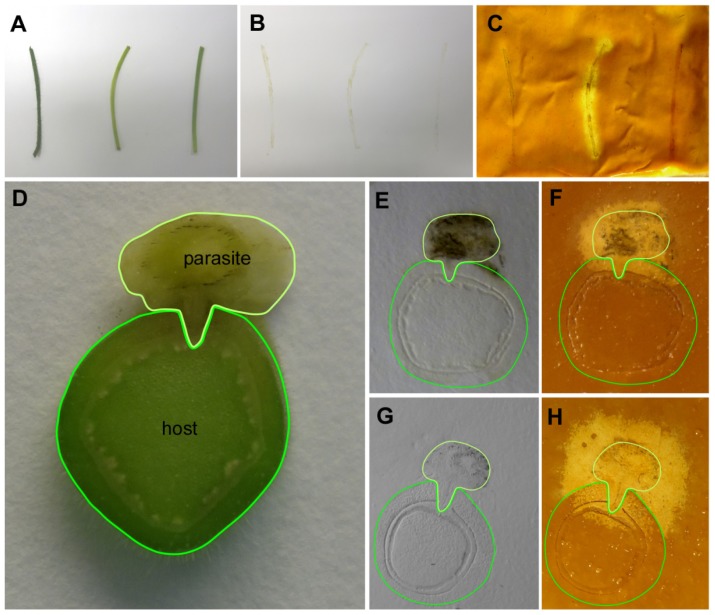
Gram’s iodine detection of cellulase activity in tissue prints covered with a layer of gelatinous CMC. (**A**–**C**) Cellulase activity in longitudinal sections prepared from stems of (from left) *Solanum lycopersicum*, *Cuscuta reflexa* and *Pelargonium zonale*; and (**D**–**H**) Localization of cellulase activity in cross-sections of parasitic plant infection sites. For ease of reference host and parasite (including the haustorium penetrating into the host) have been encircled with dark green and light green lines, respectively; (**A**,**D**) plant material as printed; (**B**,**E**,**G**) tissue prints on dry nylon membrane; and (**C**,**F**,**H**) zymograms after coating with 4% CMC and Gram’s iodine staining.

In an attempt to develop a more robust method, the gelling capacity of the substrate CMC itself was examined for its suitability for an alternative assay. At concentrations of 4% and higher, CMC produced a gel-like layer on a variety of surfaces such as, for example, on polystyrene plates or wells (Figure 2) or on nylon membranes (Figure 3). The CMC gel was stainable by Gram’s iodine, giving a reddish-brown color. Commercial cellulase as well as crude plant and soil extracts mediated a degradation of the CMC at 27 °C, leading to clearance zones when plates were stained after 12–16 h, just like on the CMC–agar plates (Figure 2A and data not shown). Agarase and amylase did not break down the CMC (Figure 2A), so that the specificity of this assay is superior to approaches using agar, agarose or Gelrite.

CMC layers with a reproducible thickness were most easily achieved in 96-well microtiter plates (Figure 2B). In these microtiter plates the degree of depolymerisation in the presence of a given amount of enzyme and the resulting retention of the iodine stain by the remaining CMC gel were highly reproducible. These plates provided the additional advantage that smaller sample volumes were necessary for getting a measurable clearance and that the digestion could be quantified with a plate reader. Figure 2C shows that there is a negative linear correlation between the staining intensity of Gram’s iodine and the amount of enzyme applied to the CMC surface when these amounts ranged between 0.2 and 100 μg of the enzyme. With 100 to 200 μg of enzyme applied per well, the curve flattened noticeably, indicating that most or all of the CMC was digested and at enzyme amounts higher than 200 μg the reaction had positively reached saturation.

The addition of up to 0.1% Tween-20 and of up to 300 mM sodium chloride to the enzyme solution did not influence the activity or the staining behavior of the assay (data not shown). The assay can therefore be employed for extracts from samples where the solubilization of the enzyme activity may require an addition of mild detergents or medium salt concentrations.

The plate- and 96-well methods described above are ideal for rapid qualitative and quantitative measurements of enzyme extracts from whole specimens or soil samples. A rapid way to quickly compare different tissues in a native context is the tissue print technique. Plant tissue prints, for example, give detailed reproductions of the tissue and its prevailing enzyme activities and are ideal for producing zymograms at organ- or tissue-level resolution [11]. The simplicity of this method makes it applicable to low or high throughput approaches (e.g., for the screening for transgenic progeny). A combination of the tissue printing technique with the cellulase detection method based on gelatinous CMC would thus be an asset for cellulase quantification in plants.

A screening of different types of membrane filters [*i.e.*, nitrocellulose, positively charged nylon and polyvinylidene difluoride (PVDF)] revealed that nylon was best compatible with the Gram’s iodine staining procedure. Purified commercial cellulase spotted in different concentrations onto a nylon filter could be detected with high specificity and sensitivity after applying a thin film of gelatinous CMC. For this, the CMC-coated membrane was incubated at 27 °C for 2 h. During this time, CMC not exposed to cellulolytic activity formed a stable and stainable gel layer, while the cellulase prevented this gel formation through the breakdown of the polymer. In these areas, Gram’s iodine failed to stain the CMC, which was, in fact, removed by the subsequent washes. As a result, a negative image emerged that matched the area where the cellulase activity was applied. Again, the assay was specific for cellulase and did not produce negative images with other enzymes such as agarase, Amylase amylase or proteinase K (data not shown). At higher cellulase concentrations, though, the enzyme activity extended beyond the actual spotting area. Thinner CMC layers were able to mitigate this effect partly, but at the expense of staining intensity in the undegraded regions (data not shown).

The parasitic species *Cuscuta reflexa* was subsequently employed to test the CMC assay with plant tissue prints. Parasitic plants of the genus *Cuscuta* have been reported to be particularly rich in cell wall degrading enzymes [12], including a highly active cellulase (see conference proceedings published in [13]). *Cuscuta* has an extremely reduced morphology and essentially lacks leaves and roots, but has developed an infection organ, the haustorium, with which it withdraws nutrients and water from its hosts. Printed non-infectious stem sections of *C. reflexa* were compared with stem-prints of two non-parasitic plants, tomato (*Solanum lycopersicum*) and *Pelargonium zonale*. In addition, cross sections through infection sites of *C. reflexa* on *P. zonale* (Figure 3) were assayed. Cellulase activity was strongly detected in the longitudinal sections through the stem of *C. reflexa*. In contrast, neither the stems of tomato (*S. lycopersicum*) nor of *P. zonale* showed any detectable degradation of the CMC (Figure 3A–C). Tissue prints of cross sections from *P. zonale* infected by *C. reflexa* (Figure 3D–H) also showed that the cellulase activity is mainly confined to the stem of the parasite. Host stem areas closest to the parasite stained as well, but the tendency of high enzyme activities to spread beyond the borders of the actual tissue print (see text above and Figure 3F,H) made it unfortunately difficult to judge whether this activity in the infected host was attributable to an elevated host enzyme activity in this region, a secreted cellulase activity from *C. reflexa*, or whether it simply was due to an unspecific halo from the stem tissue of the parasite. Crosslinking the printed proteins to the membrane might solve this problem and increase the spatial resolution, but also poses the danger that some of the activity might be compromised by this treatment.

The high cellulolytic activity in the infection organs of the parasite can be explained by the necessity of the parasite to penetrate the host tissue, a process that is highly dependent on secreted enzymes. More surprising, however, was that also the longitudinal stem sections that did not contain any infective organs produced a strong reaction. A possible explanation might be an elevated longitudinal growth of *Cuscuta* stems above the infective area that could necessitate a constant restructuring of its cell walls, which in turn would require the activity of cellulases to break up the otherwise rigid structure of the cell wall.

In summary, we found that pure CMC (without agar or other gel-forming polymers) is both specific and sensitive enough to indicate the presence of cellulolytic activity in soluble extracts and in tissue prints of selected plants. Although this has not been tested, the methods shown here should be easily transferable to screen colony-forming microorganisms by applying colony lifts from agar-plates onto nylon membranes and coating these with a layer of CMC.

## 3. Experimental Section

### 3.1. Agar Plate-Based Clearing Assays

1% agar (catalog number A1296, Sigma-Aldrich Corp., St. Louis, MO, USA) containing 0.2% carboxymethylcellulose (catalog number C4888, Sigma-Aldrich Corp., St. Louis, MO, USA) was poured into petri dishes or into the wells of a 96-well plate, respectively. Stock solutions of polymer-degrading enzymes were dissolved in 1× Tris-buffered saline (TBS) buffer or deionized water, following the suppliers’ instructions for reconstitution of the enzyme. Spot plating was performed with 5 μL of commercial cellulase from *Aspergillus niger* (catalog number C1184, Sigma-Aldrich Corp., St. Louis, MO, USA) at different concentrations (0.1 and 5 μg/μL). Agarase (1 μg/μL) (catalog number EO0461, Fermentas, Burlington, ON, Canada), amylase (1 μg/μL) (catalog number 10065, Sigma-Aldrich Corp., St. Louis, MO, USA) and proteinase K (1 μg/μL) (catalog number EO0491, Fermentas, Burlington, ON, Canada) were used as control enzymes. In some cases, agar was replaced by 1% agarose or 0.75% Gelrite with or without 0.2% CMC, respectively. All plates were incubated at 27 °C for 12–16 h after which hydrolysis zones were visualized by flooding of the plates/wells with Gram’s iodine (2 g potassium iodide and 1 g iodine in 300 mL water) for 5 min followed by a rinses with deionized water. For detection with Congo Red, the plates were flooded with 0.1% Congo Red for 15–20 min and then rinsed with 1 M NaCl. Plates where CMC was omitted were used as non-substrate controls in all experiments.

### 3.2. Pure, Solidified CMC Plate-Based Clearing Assays

A solution of 7% CMC was heated to 70 °C, poured into petri dishes and was allowed to polymerize at room temperature overnight. Spot plating was performed with 5 μL of cellulase (1 μg/μL), amylase (1 μg/μL) and agarase (1 μg/μL), followed by an incubation at 27 °C for 12–16 h. Hydrolysis zones were visualized by flooding of the plates with Gram’s iodine as described above. For assays in 96-well plates, 200 μL 4% CMC was pipetted into the plate wells and allowed to polymerise at room temperature for 12–16 h. Twenty microliter of cellulase from *A. niger* containing the indicated amounts of the enzyme (see Figure 2) were added to each well. The plate was incubated at 27 °C for 2 h after which hydrolysis was visualized by flooding the wells with Gram’s iodine for 20 min. Following removal of the staining solution, the plates were washed twice with 20% ethanol. The absorption at 575 nm as a result of staining was measured photometrically in a microplate reader.

### 3.3. Tissue Prints

Longitudinal or cross sections freshly prepared from stems of *Solanum lycopersicum*, *Cuscuta reflexa* and *Pelargonium zonale* were printed onto a positively charged nylon membrane filter (Roche, Indianapolis, IN, USA) for 10–15 s using moderate pressure. After a short drying period, the prints were covered evenly with approximately 3 mL of 4% CMC per 100 cm^2^ of membrane. The coated membrane was incubated for 2 h at 27 °C. Staining with Gram’s iodine was carried out for 5 min. To differentiate the digested area on the prints, the membrane was briefly rinsed once or twice in water to remove excess staining solution that was not absorbed by the CMC gel. This was followed by a brief washing step in 70% EtOH and a second, longer wash (~2 min) in 70% EtOH. Results were recorded digitally using a conventional camera while the prints were still wet.

## 4. Conclusions

Pure, solidified CMC is a reliable substrate for relative comparisons and quantitative assessments of cellulase activities that correlate with published activities. Thin CMC-layers can be used to screen for cellulase activities in plant tissue prints. These tissue print zymograms can facilitate the detection of high cellulolytic activities in plants from different habitats or in transgenic plants engineered for optimized cell wall degradation without the interference of intrinsic sugars.
